# Client-provider interactions in provider-initiated and voluntary HIV counseling and testing services in Uganda

**DOI:** 10.1186/1472-6963-13-423

**Published:** 2013-10-19

**Authors:** Rhoda K Wanyenze, David Kyaddondo, John Kinsman, Fredrick Makumbi, Robert Colebunders, Anita Hardon

**Affiliations:** 1 Department of Disease Control and Environmental Health, Makerere University School of Public Health, P.O. Box 7072, Kampala, Uganda; 2 Makerere University Department of Social Work/Child Health Development Centre, Kampala, Uganda; 3 Umeå Centre for Global Health Research, Department of Public Health and Clinical Medicine, Umeå University, Umeå, Sweden; 4 Institute of Tropical Medicine, Antwerp, Belgium; 5 University of Amsterdam, Amsterdam, Netherlands

## Abstract

**Background:**

Provider-initiated HIV testing and counselling (PITC) is based on information-giving while voluntary counselling and testing (VCT) includes individualised client-centered counseling. It is not known if the provider-client experiences, perceptions and client satisfaction with the information provided differs in the two approaches.

**Methods:**

In 2008, we conducted structured interviews with 627 individuals in Uganda; 301 tested through PITC and 326 through voluntary counselling and testing (VCT). We compared client experiences and perceptions based on the essential elements of consent, confidentiality, counseling, and referral for follow-up care. We conducted multivariate analysis for predictors of reporting information or counselling as sufficient.

**Results:**

In VCT, 96.6% (282) said they were asked for consent compared to 91.3% (198) in PITC (P = 0.01). About the information provided, 92.0% (286) in VCT found it sufficient compared to 78.7% (221) in PITC (P = <0.01). In VCT 79.9% (246) thought their results were kept confidential compared to 71.7% (200) in PITC (P = 0.02). Eighty percent (64) of HIV infected VCT clients said they were referred for follow-up care versus 87.3% (48) in PITC (p = 0.2). Predictors of perceived adequacy of information in PITC included an opportunity to ask questions (adj.RR 1.76, CI 1.41, 2.18) and expecting the test results received (adj.RR 1.18, CI 1.06, 1.33). For VCT significant factors included being given an opportunity to ask questions (adj.RR 1.62, CI 1.00, 2.60) and 3+ prior times tested, (adj.RR 1.05, CI 1.00, 1.09).

**Conclusions:**

This study demonstrates good practices in the essential elements of HIV testing for both VCT and PITC. However, further quality enhancement is required in both testing approaches in relation to referral to HIV care post-test, client confidence in relation to confidentiality, and providing an opportunity to ask questions to address client-specific information needs.

## Background

HIV counselling and testing (HCT) is critical to the expansion of access to HIV prevention, care and treatment services [1]. In addition to several emerging prevention tools, treatment as prevention has gained prominence with introduction of life-long treatment for HIV infected pregnant women and increasing CD4 cut-off for initiation of antiretroviral treatment for adults and adolescents [2-5]. All these interventions are hinged on large scale testing to identify and treat people who are HIV infected in order to reduce the risk of HIV transmission.

In order to scale up access to HCT alternative approaches to HCT including home-based and provider-initiated HIV testing and counselling (PITC) have been widely adopted in many countries [6-9]. Several studies and systematic reviews show increased uptake of HCT and access to services following introduction of PITC [10-12]. In Uganda PITC was adopted in 2005 with a focus on diagnosing and linking HIV infected individuals to care and treatment [9,12]. Counseling pre-test was reduced to introducing the HIV test and explaining the patient’s right to opt-out as well as options for care if they are HIV infected. Pre-test counseling on risk-reduction was no longer required [13]. The didactic post-test session highlights prevention options, disclosure and partner testing [13]. On the other hand, the VCT protocol continues to demand detailed client-centered HIV risk assessment and risk reduction counseling before and after the test [9].

Consent, confidentiality, adequate counseling or information, and referral for other relevant services post-test are critical elements of good quality HCT. There are clear differences between VCT and PITC however, ensuring quality of HCT services irrespective of the approach remains critical. The aim of this study was to compare the client experiences and perceptions of quality as well as satisfaction with counseling or information provided in VCT and PITC.

We conducted structured interviews with clients tested through VCT and PITC in Uganda, and compared their experiences and perceptions of consent, counselling and confidentiality. This study was done as part of the Multi-Country African Study on Testing and Counselling for HIV (MATCH) which investigated HIV testing and counselling practices and experiences in four African countries: Burkina Faso, Kenya, Malawi, and Uganda [14]. The study was approved by the Makerere University Child Health Development Center Ethics Committee and the Uganda National Council for Science and Technology.

## Methods

Between May and December 2008, we conducted structured client interviews with 627 adults (≥18 years) including 301 tested through PITC and 326 tested through VCT.

### Study sites

At the time of conducting this study, PITC (outside PMTCT) in Uganda was implemented largely with support from Mulago-Mbarara Teaching Hospitals’ Joint AIDS Program (MJAP) and Research Triangle International (RTI). We selected facilities supported by each of the two programs. The selected sites included the national referral and teaching hospital (Mulago Hospital), a regional referral hospital (Soroti Hospital) and a level IV Health Centre (Mpigi Health Center). All the three sites had implemented PITC for over two years. For VCT, we selected the largest and longest VCT provider in Uganda (AIDS Information Center), including an upcountry site in Soroti and another in the capital city of Kampala.

### Selection of respondents

Within the health facilities patients were selected from medical and TB wards, out-patient and antenatal clinics. Sampling and selection of respondents varied by site and client load. The Mulago antenatal care (ANC) had many clients, so we selected every 5th person every clinic day. On the other hand, the wards in the regional referral hospital and HC IV did not have as many patients so all patients who were discharged on any given day were approached for participation. Similarly, all TB patients who were discharged from the wards were approached for participation. For the VCT facilities we selected every 3rd client on a daily basis. Respondents at the VCT sites and PITC outpatient clinics were approached for participation in the waiting area but interviews were conducted after their session with the providers (on exit from the clinics). Inpatients were approached and interviewed immediately after discharge from the wards.

All respondents provided written consent for participation (signature or thumb print). Out of 303 individuals who were approached within PITC, all agreed to be interviewed but two did not complete the interview. Out of 340 VCT clients who were approached for participation, 4 declined and 10 who had initially agreed to participate did not complete the interview. The major reason for decline and failure to complete interviews was lack of time. Clients were rushing home after being discharged or getting their HIV results.

### Measures

The structured interviews elicited information on client experiences with consent and counseling and perceptions of confidentiality of their test results.

Measures of consent were: whether providers asked clients for consent before testing, and whether providers informed them that they had a right to agree or decline testing.

In addition, we asked whether the PITC clients thought it was important to be given a chance to consent before testing. Measures for confidentiality were: whether providers told clients that their test results would be kept confidential, and clients’ perceptions of whether they felt their results would be kept confidential. In terms of adequacy of counselling, we used a standardised protocol of information that should be given during pre- and post-test counselling based on the Uganda national VCT protocol which includes: how HIV is transmitted; how the test works; the meaning of positive and negative results; explanation of the window period; information on prevention, disclosure and partner testing. In terms of overall perceived quality of the counselling and testing services, we asked if the clients felt the information they had received was sufficient, whether they were given an opportunity to ask questions, how they were treated by providers, and if they found the interaction with the providers useful. The same questions were asked of respondents in PITC and stand-alone VCT.

### Data analysis

We compared experiences across the two strategies and used chi-square tests to test for differences across the two groups. Within PITC we conducted a sub-analysis and comparisons of respondents including in ANC, medical inpatients, and outpatients. TB inpatients were combined with other medical inpatients since the number of TB inpatients interviewed was small (<30). We conducted bivariate and multivariate analysis for factors associated with perceived adequacy of information or counseling. We assumed that there would be differences by age, marital status, educational level and sex; and also by HIV sero-status, whether clients expected the results they received and if they were given an opportunity to ask questions. The outcome variable for the bivariate and multivariate analysis was proportion of clients reporting that information received was sufficient. Because of the inherent differences in testing approach between VCT (client-initiated) and PITC (provider-initiated), we developed separate models for PITC and VCT. The model of choice was a generalized linear model (GLM) with the binomial family and a log link regression but because of convergence problems with this model, we opted for a “modified” Poisson model (under GLM with Poisson family and log link with robust standard errors with out exposure offset) and adjusted for clustering at geographical location of health facility (Soroti, Mulago/Kampala and Mpigi) to obtain prevalence risk ratio (RR) of reporting that information provided was sufficient[15]. In the multivariate analysis, we included variables from the bivariate analysis with p-value < 0.15 or potential confounders. Statistical analyses were performed using Stata™ Release 9.2 (Stata Corporation, 4905 Lakeway Drive, College Station, Texas 77845 USA).

## Results

More respondents were women than men: 53.4% (174) in VCT and 62.1% (187) in PITC. VCT respondents were more often never married, 44.8% (145) compared to 18.4% (55) in PITC, p < 0.0001; and a larger proportion of respondents in VCT, 50.3% (157) were first-time testers compared to 39.0% (112) in PITC (Table 1).

**Table 1:** Socio-demographic characteristics of respondents

**Respondent characteristics**	**Total N = 627**	**VCT N (%) N = 326**	**PITC N (%) N = 301**
**Age**			
Mean (SD)	31.26 (10.72)	30.2 (8.85)	32.5 (12.36)
Median (IQR)	29 (24, 37)	28 (24, 35)	29 (24,38)
**Age-group**			
18-20	76 (12.2)	40 (12.3)	36 (12.0)
21-30	302 (48.3)	162 (49.7)	140 (46.8)
31-40	151 (24.2)	81 (24.9)	70 (23.4)
41-50	58 (9.3)	33 (10.1)	25 (8.4)
50+	38 (6.1)	10 (3.1)	28 (9.4)
**Sex**			
Female	361 (57.6)	174 (53.4)	187 (62.1)
Male	266 (42.4)	152 (46.6)	114 (37.9)
**Education**			
No formal education	39 (6.3)	21 (6.5)	18 (6.0)
Primary	226 (36.2)	79 (24.3)	147 (49.2)
Secondary/vocational	282 (45.2)	166 (51.1)	116 (38.8)
Post-secondary	71 (11.3)	56 (17.2)	15 (5.0)
Other	6 (1.0)	3 (1.0)	3 (1.0)
**Marital status**			
Never married	200 (32.1)	145 (44.8)	55 (18.4)
Married or cohabiting	327 (52.5)	129 (39.8)	198 (66.2)
Divorced/separated	55 (8.8)	27 (8.3)	28 (9.4)
Widowed	41 (6.6)	23 (7.1)	18 (6.0)
**Religion**			
Catholic	205 (32.7)	89 (27.3)	116 (38.5)
Protestant	222 (35.4)	127 (39.0)	95 (31.6)
Moslem	102 (18.3)	62 (19.0)	40 (13.3)
Pentecostal	84 (13.4)	41 (12.6)	43 (14.3)
SDA	14 (2.2)	7 (2.2)	7 (2.3)
**Previous HIV testing**			
Once	269 (44.9)	157 (50.3)	112 (39.0)
Twice	137 (22.9)	57 (18.3)	80 (27.9)
Thrice	193 (32.2)	98 (31.4)	95 (33.1)

### Consent process

The majority of respondents in VCT (82.3%, 256) and PITC (83.9%, 239) said they did not find it difficult to decide to be tested. The majority of respondents also reported that they were asked if they agreed to be tested; significantly higher in VCT, 96.6% (282) than PITC, 91.3% (198), (p = 0.01). A lower proportion said they were told about their right to decline testing; again this measure of consent was higher in VCT, 89.7% (262) than PITC, 80.1% (173), (p = 0.002). All respondents who were asked for consent said they agreed to be tested except one in VCT and nine in PITC who said they did not know. When PITC respondents were asked how important it was for them to agree or decline to be tested majority (65.5%, 116) said it was very important, 20.3% (36) said it was somewhat important while the rest thought it was not important.

### Receipt of results and confidentiality

Almost all respondents reported that they had received their results; 99.4% (313) in VCT and 97.2% (280) in PITC (P = 0.1). However three VCT respondents declined to share their results. Among those who shared their results, 27.0% (88) in VCT and 20.1% (56) in PITC said they were HIV infected. Within PITC 26.9% inpatients reported that they were HIV positive compared to 16.9% outpatients and 14.5% in ANC. A significant proportion of the respondents said they were not expecting the results they received in terms of the HIV sero-status; 36.0% (111) in VCT and 37.8% (105) in PITC (P = 0.05).

The majority of the clients were told by providers that their results would be kept confidential; 94.5% (274) in VCT compared to 85.3% (185) in PITC (P = 0.007). Asked whether they felt their results had been protected so that nobody else knows them, the majority agreed; 79.9% (246) in VCT versus 71.7% (200) in PITC (P = 0.02). A significant proportion of the clients said they did not know whether this had happened; 19.5% (60) in VCT and 25.5% (71) in PITC. In terms of whether the clients thought confidentiality was important, 73.1% (228) in VCT agreed compared to 67.6% (188) in PITC (P = 0.5).

### Pre- and post-test counselling

The majority of respondents in VCT and PITC received pre-test information on how HIV is transmitted, how the test works, the meaning of positive and negative results, explanation of the window period, and information on prevention, and we did not detect any significant differences by strategy (Table 2). During the post-test session, they received the same information but the PITC were less likely to receive the information (Table 2). A high proportion of respondents across both strategies said the information they received was sufficient but this was significantly lower in PITC; 92.0% (286) in VCT versus 78.7% (221) in PITC (P = <0.001). About the opportunity to ask questions, 96.1% (297) in VCT agreed compared to 71.5% (201) in PITC (p < 0.001). On how useful the meeting with the providers was, 99.4% (307) in VCT said it was useful compared to 86.8% (244) in PITC (p < 0.001). The majority of respondents said they were treated well by the providers (Table 2). Eighty percent (64) of the HIV infected individuals in VCT and 87.3% (48) from PITC said they were referred for follow-up care (p = 0.3).

**Table 2:** Client perceptions of the confidentiality, counselling and consent procedures within VCT and PITC

**Client perceptions/experiences**	**Total N = 627**	**VCT N = 326**	**PITC N = 301**	**Chi square (p value)**
***Pre-test session***				
**Asked if they agreed to test**				
Yes	480 (94.3)	282 (96.6)	198 (91.2)	
No	29 (5.7)	10 (3.4)	19 (8.8)	0.010
**Explained choice to decline test**				
Yes	435 (85.6)	262 (89.7)	173 (80.1)	0.002
No	73 (14.4)	30 (10.3)	43 (19.9)	
**Told that results would remain confidential**				
Yes	459 (90.2)	274 (93.8)	185 (85.3)	
No	48 (9.4)	16 (5.5)	32 (14.8)	0.001
**Explained HIV transmission**				
Yes	456 (89.6)	267 (91.4)	189 (87.1)	
No	52 (10.2)	24 (8.2)	28 (12.9)	0.159
**Explained how the test works**				
Yes	365 (71.9)	212 (72.6)	153 (70.8)	
No	143 (28.2)	80 (27.4)	63 (29.2)	0.661
**Explained + ve/-ve results**				
Yes	461 (91.3)	268 (92.7)	193 (89.4)	
No	44 (8.7)	21 (7.3)	23 (10.7)	0.182
**Explained window period**				
Yes	421 (82.7)	252 (86.3)	169 (77.9)	
No	85 (16.7)	39 (13.4)	46 (21.2)	0.071
**Given advice on prevention**				
Yes	483 (94.9)	281 (96.2)	202 (93.1)	
No	26 (5.1)	11 (3.8)	15 (6.9)	0.111
***Post-test session***				
**Explained meaning of results**				
Yes	569 (96.0)	309 (99.0)	260 (92.5)	
No	21 (3.5)	3 (1.0)	18 (6.4)	0.001
**Discussed disclosure**				
Yes	395 (66.6)	221 (70.8)	174 (61.9)	
No	193 (32.6)	90 (28.9)	103 (36.7)	0.076
**Discussed prevention**				
Yes	537 (90.6)	302 (96.8)	235 (83.6)	
No	51 (8.6)	10 (3.2)	41 (14.6)	<0.001
**Referral for medical care**				
Yes	112 (83.0)	64 (80.0)	48 (87.3)	0.2690
No	23 (17.0)	16 (20.0)	7 (12.7)	
***Overall session (Perceived quality)***				
**Information given sufficient**				
Yes	507 (85.6)	286 (92.0)	221 (78.7)	
No	57 (9.6)	15 (4.8)	42 (15.0)	<0.001
Do not know	25 (4.2)	9 (2.9)	16 (5.7)	
**Felt results were kept confidential**				
Yes	446 (76.0)	246 (79.9)	200 (71.7)	
No	10 (1.7)	2 (0.7)	8 (2.9)	0.020
Don’t know	131 (22.3)	60 (19.5)	71 (25.5)	
**Opportunity to ask questions**				
Yes	498 (84.4)	297 (96.1)	201 (71.5)	
No	85 (14.4)	12 (3.9)	73 (26.0)	<0.001
**How they were treated by providers**				
Very well	243 (41.0)	144 (46.2)	99 (35.2)	0.021
Well	337 (56.8)	167 (53.6)	170 (60.5)	
badly	5 (0.8)	1 (0.3)	4 (1.4)	
Declined to answer	8 (1.4)	0(0)	8(2.9)	
**Expected results they received**				
Yes	336 (57.2)	188 (60.8)	148 (53.2)	
No	216 (36.8)	111 (35.9)	105 (37.8)	0.017
Don’t know	33 (5.6)	9 (2.9)	24 (8.6)	

### Experiences of antenatal, inpatients and outpatients

Within PITC the experiences of patients were similar across the clinics and wards but there were differences for some parameters. All (73) the ANC and almost all the inpatients (95.5%, 70) reported that they had been asked if they agreed to test compared to 77.0% (52) of the outpatients. A higher proportion in ANC (94.1, 80) reported receiving HIV prevention information compared to inpatients (81.4%, 83) and outpatients (79.8%, 67). Similarly, a higher proportion in ANC (88.3%, 75) reported receiving information on partner testing compared to inpatients (76.5%, 78) and outpatients (78.6%, 66). Among ANC clients, 98.6% (72) reported that they were given an opportunity to ask questions, compared to 90.3% of inpatients and 89.7% (62) of outpatients. A lower proportion across all the three categories of patients reported that the information they received was sufficient; 86.6% (71) in ANC compared to 80.0% (80) of inpatients and 86.8% (66) of outpatients.

### Predictors of sufficient information or counselling

In the bivariate analysis for PITC, individuals who received results that they expected were more likely to find the information sufficient (RR 1.21, CI 1.06-1.37). Also, individuals who were given an opportunity to ask questions were more likely to find the information sufficient (RR 1.73, CI 1.39-2.16). There were no significant differences by type of patients (ANC, inpatients and outpatients), number of previous HIV tests, HIV sero-status, gender and age (Table 3). In the multivariate analysis for PITC, significant predictors of information being sufficient included being given an opportunity to ask questions (adj.RR 1.73, CI 1.43-2.10) and expecting the results they received (adj.RR 1.07, CI 1.01-1.14). Individuals who had previously tested for HIV once or twice were less likely to report that the information was sufficient while those who had tested three or more times were not significantly different from those who were testing for the first time (Table 3).

**Table 3:** Proportion, crude and adjusted risk ratio for association between reporting that counseling information was sufficient by PITC respondent’s characteristics

	**Total respondents**	**Number reporting information was sufficient**	**Percentage reporting information was sufficient**	**Crude risk ratio (95% CI)**	**Adjusted risk ratio (95% CI)**
	**N**	**'n**	**100, (%)**		
**Overall**	265	213	80.4		
**Age (years)**					
18-20	32	29	90.6	1.0	
21-30	128	103	80.5	***0.89 (0.79, 0.997)***	
31-40	63	48	76.2	0.84 (0.67, 1.06)	
41-50	21	17	81.0	0.89 (0.75, 1.06)	
51+	21	16	76.2	0.84 (0.62, 1.14)	
**Sex**					
Female	166	134	80.7	1.0	1.0
Male	99	79	79.8	0.99 (0.85, 1.15)	1.00 (0.85, 1.17)
**Times tested for HIV**					
1	103	86	83.5	1.0	1.0
2-3	124	96	77.4	0.93 (0.77, 1.12)	***0.88 (0.83, 0.94)***
4+	38	31	81.6	0.98 (0.76, 1.25)	0.99 (0.76, 1.30)
**Test results expected**					
No/do not know	123	89	72.4	1.0	1.0
Yes	142	124	87.3	***1.21 (1.16, 1.25)***	***1.18 (1.10, 1.28)***
**Reported HIV status**					
Negative/Indeterminate	211	169	80.1	1.0	1.0
Positive	54	44	81.5	1.02 (0.84, 1.23)	0.96 (0.83, 1.12)
**Opportunity to ask**					
No/do not know	74	39	52.7	1.0	1.0
Yes	191	174	91.1	***1.73 (1.52, 1.96)***	***1.76 (1.43, 2.16)***
**Type of Facility**					
ANC	83	70	84.3	1.0	1.0
In-patient	101	78	77.2	1.05 (0.77, 1.44)	0.93 (0.67, 1.30)
OPD	81	65	80.2	0.96 (0.80, 1.15)	0.96 (0.82, 1.17)

Within VCT, receiving results that they expected and being given an opportunity to ask questions were not significantly associated with information being sufficient at bivariate analysis (Table 4). There were also no significant differences by HIV sero-status, gender and age. However the number of previous HIV tests was significant; individuals who had previously tested for HIV once or twice were less likely to find the information sufficient while those who had previously tested for three or more times were more likely to find the information sufficient (Table 4). In the multivariate analysis for VCT, significant predictors of information being sufficient included being given an opportunity to ask questions (adj.RR 1.62, CI 1.00-2.60) and the number of previous tests (Table 4).

**Table 4:** Proportion, crude and adjusted risk ratio for association between reporting that counseling information was sufficient by VCT respondent’s characteristics

	**Total respondents**	**Number reporting information was sufficient**	**Percentage reporting information was sufficient**	**Crude risk ratio (95% CI)**	**Adjusted risk ratio (95% CI)**
	**N**	**'n**	**100, (%)**		
**Overall**	300	277	92.3		
**Age (years)**					
18-20	33	30	90.9	1.0	
21-30	149	138	92.6	1.02 (0.97, 1.07)	
31-40	76	70	92.1	1.01 (0.996, 1.03)	
41-50	32	29	90.6	1.00 (0.89, 1.11)	
51+	10	10	100.0	1.10 (0.99, 1.22)	
**Sex**					
Female	159	144	90.6	1.0	1.0
Male	141	133	94.3	1.04 (0.98, 1.11)	1.03 (0.98, 1.08)
**Times tested for HIV**					
1	150	141	94.0	1.0	1.0
2-3	98	84	85.7	***0.91 (0.88, 0.94))***	***0.90 (0.89, 0.91)***
4+	52	52	100.0	1.06 (0.95, 1.18)	1.05 (0.94, 1.17)
**Test results expected**					
No/do not know	118	109	92.4	1.0	1.0
Yes	182	168	92.3	1.00 (0.99, 1.00)	0.99 (0.98, 1.01)
**Reported HIV status**					
Negative/Indeterminate	220	205	93.2	1.0	1.0
Positive	80	72	90.0	***0.97 (0.95, 0.99)***	***0.96 (0.94, 0.98)***
**Opportunity to ask**					
No/do not know	12	7	58.3	1.0	1.0
Yes	288	270	93.8	***1.61 (0.95, 2.72)***	1.62 (0.97, 2.69)

## Discussion

These data show good practices in relation to the essential elements of HIV testing in both VCT and PITC. The majority of the PITC respondents thought it was important to decline or accept testing. However, some did not think it was important. This may be associated with the illness and need for diagnosis among PITC respondents and for the ANC the moral issue of protecting the unborn child.

The interaction and information giving within PITC is shorter to enable integration within busy health facilities and it seems to work well for the majority of the patients [6,9,16,17]. There were a few individuals in both PITC and VCT who did not find the information adequate. We found that individuals who receive results that they do not expect in PITC need more support than others. The results also indicate that giving the clients an opportunity to ask questions in both VCT and PITC increases the likelihood of finding the information provided to be adequate. This is not surprising because the dialogue would lead to clients raising issues that the providers may overlook and highlights the need to closely examine the practices around information giving to ensure that the patients have an opportunity to raise concerns.

Within VCT an individual has thought about testing and made a deliberate decision to go for the test. For PITC on the other hand, depending on the nature of illness, a patient may come to hospital when they have not thought about HIV testing. This may explain why receiving results that were not expected was more important in PITC than VCT. For home-based HIV testing, another provider-initiated testing strategy, it has been reported that the social mobilization that precedes testing increases acceptance and uptake of testing [18-20]. Social mobilization efforts within countries that have adopted PITC may be necessary to ensure that people are aware and expect PITC when they go to health facilities.

A significant proportion of respondents in both strategies had some doubts about confidentiality. This study did not evaluate why the clients felt this way. However, the data highlights the need to address confidentiality concerns among clients.

Although the Ugandan PITC guidelines recommend risk reduction information post-test and not pre-test, to reduce on the time required for HCT, in practice providers gave this information both pre- and post-test and there was little difference between PITC and VCT in the pre-test information.

This study compared clients who were interviewed soon after testing within PITC and VCT. The clients’ perceptions of the testing may change over time depending on their coping ability and experiences after the test. However, the study highlights important quality issues that could be improved in both VCT and PITC. Clients who do not get the result that they expect, and those who come for repeat testing may need more attention post-test. Overall clients value being asked whether they have questions.

## Conclusions

Overall, the quality of HCT was good in both strategies, in relation to the essential elements of HIV testing. However, the findings show a need for further quality enhancement in both testing approaches in relation to referral to HIV care post-test, client confidence in relation to confidentiality, and providing an opportunity to ask questions to address client-specific information needs.

## Competing interests

The authors declare that they have no competing interests.

## Authors’ contributions

RKW initiated the topic and wrote the first draft of the paper. DK, RC and AH contributed to the design of the topic, interpretation of findings and writing of the paper. FM analysed the data and contributed to the interpretation of the findings. JK contributed to the interpretation of findings and writing of the paper. All authors read and approved the final manuscript.

## Pre-publication history

The pre-publication history for this paper can be accessed here:

http://www.biomedcentral.com/1472-6963/13/423/prepub
